# Regional analgesia techniques for effective recovery from coronary artery bypass surgeries: a retrospective study involving the experience of a single center

**DOI:** 10.1186/s13019-022-01923-6

**Published:** 2022-07-06

**Authors:** Sami Kaan Cosarcan, Özer Ali Sezer, Sami Gürkahraman, Ömür Erçelen

**Affiliations:** 1 Department of Anesthesiology, VKV American Hospital, Istanbul, Turkey; 2 Department of Anesthesiology and Pain Clinic, VKV American Hospital, Istanbul, Turkey; 3 Department of Cardiovascular Surgery, VKV American Hospital, Istanbul, Turkey

**Keywords:** Regional anesthesia, Fascial plane blocks, Cardiac surgeries, Coronary artery bypass surgery, ERAS, Postoperative analgesia, Early extubation, Fast track recovery

## Abstract

**Background:**

Pain after cardiac surgery is both multifocal and multifactorial. Sternotomy, sternal retraction, internal mammary dissection, posterior rib dislocation or fracture, potential brachial plexus injury, and mediastinal and pleural drains all contribute to pain experienced in the immediate postoperative period. Ineffective pain management can result in systemic and pulmonary complications and significant cardiac consequences.

**Methods:**

This study compared the effectiveness of regional anesthesia techniques for perioperative pain management in cardiac surgery patients at our clinic. The effects of different analgesic methods, in terms of contributing to recovery, were examined.

**Results:**

The records of 221 patients who had undergone coronary bypass surgery were evaluated retrospectively. The extubation rate in the operating room was 91%. No patient received balloon pump support, and 20 patients were transferred to the cardiovascular intensive care unit while intubated. Regional anesthesia was performed on two of these 20 patients, but not on the remaining 18. Examination of intraoperative and postoperative opioid consumption revealed significantly lower levels among patients receiving regional anesthesia. The most effective results among the regional anesthesia techniques applied were achieved with double injection erector spinae plane block.

**Conclusion:**

Regional anesthesia techniques severely limit opioid consumption during cardiac surgery. Their importance will gradually increase in terms of rapid recovery criteria. Based on our study results, double injection of the erector spinae plane block seems to be the most effective technique in cardiac surgery. We therefore favor the use of fascial plane blocks during such procedures.

*Trial Numbers* The study is registered with ClinicalTrials (NCT05282303). Ethics committee registration and approval were Granted under Number 2021.464.IRB1.131.

## Introduction

Definitive coronary bypass, traditionally performed via median sternotomy, can be associated with a significant level of surgical soft tissue and bony injury during the dissection phase. Significant pain is experienced after cardiac surgery, with 30–75% of patients reporting moderate-to-severe acute pain. Post-sternotomy patients may develop chronic pain syndrome. Higher levels of pain and greater analgesic requirements in the immediate postoperative period correlate strongly with the development of chronic pain syndrome. Pain after cardiac surgery is both multifocal and multifactorial. Sternotomy, sternal retraction, internal mammary dissection, posterior rib dislocation or fracture, possible brachial plexus injury, and mediastinal and pleural drains all contribute to pain experienced in the immediate postoperative period. A shift in pain modulation also occurs in the peripheral and central nervous systems in the hours and days following surgery. Some changes are derived from DNA methylation and the subsequent gene expression of proteins that modulate opioid analgesia. In other cases, the inflammatory cascade induces gene-driven expression of inflammatory pain modulators in the spinal cord. Pain management schemes must take into account the cause, location, and timing of pain foci in order to achieve maximal success [1, 2].

Ineffective pain management can lead to systemic and pulmonary complications and significant cardiac consequences. Tachycardia and hypertension attacks, occurring as a secondary response to pain, increase myocardial oxygen consumption and may cause hemodynamic impairment [3].

Increasing restrictions on human and financial resources in the health care system have also encouraged the use of regional anesthetic techniques as part of an opioid-sparing, multimodal pain regimen during pediatric and adult cardiac surgery for early extubation, reducing postoperative complications, minimizing the length of ICU and hospital stay, and reducing the overall cost of perioperative care. Growing advocacy for minimal or opioid-minimizing enhanced recovery protocols in perioperative settings has also been observed. All these factors have led to the adoption of multimodal analgesia in perioperative care. Neuraxial and peripheral nerve blocks are essential components of multimodal analgesic protocols [4, 5]. Thoracic epidural analgesia (TEA) is the gold standard for cardiac surgery. Its advantages include fewer cardiovascular events (stroke and myocardial ischemia) and respiratory complications, a decreased incidence of renal failure, lower infection rates, shorter ICU stays, decreased anesthesia costs, and earlier hospital discharge. Multiple clinical trials have confirmed the safety of thoracic epidurals. However, some concerns over spinal and epidural hematomas (and other potential complications) have hindered its widespread acceptance and represent an ongoing controversy. Additionally, the risk of post-cardiopulmonary bypass coagulopathy further complicates the use of neuraxial techniques. Concurrent aspirin use with systemic heparinization is a risk factor for epidural hematoma after neuraxial instrumentation. With minimally invasive cardiac surgery, fast track anesthesia techniques and multimodal analgesia, including regional anesthesia techniques, have focused on blocking peripheral nerves in neural planes under ultrasonic guidance. Chest wall blocks are more recent and easier alternatives to neuraxial analgesia [6].

This study was conducted to compare the effectiveness of regional anesthesia techniques for perioperative pain management in patients undergoing cardiac surgery at our clinic. It also examined the effects of different analgesic methods, in terms of contributing to recovery.

## Materials and methods

This study was approved by the Koç University Clinical Research Ethics Committee (2021.464.IRB1.131). Patients who underwent coronary artery bypass grafting (CABG) surgery at the VKV American Hospital, Turkey, between January 2015 and May 2020 were reviewed retrospectively. Patients with a history of cerebrovascular events, scheduled carotid surgery, or emergency CABG surgery were excluded from the study. Demographic data from the preoperative evaluation forms and operation types from the surgery reports were recorded. Intraoperative anesthesia follow-up forms were assessed, and the use or otherwise of regional anesthesia and the type and extent of opioid use during the operation were examined. Postoperative transfer forms to the cardiovascular intensive care unit (CICU), intensive care follow-up forms, and departmental ward record follow-up forms were examined. Pain scores (NRS) during rest and coughing, vital signs, mobilization, and initiation of respiratory exercises were recorded for postoperative respiratory complications.

### Exclusion criteria

Patients with a history of cerebrovascular events, Alzheimer's disease, or dementia, with inadequate cognitive functions, a history of chronic pain, or in receipt of long-term opioid therapy were excluded from the study.

### Statistical analysis

Statistical analysis was performed on using IBM SPSS Statistics for Windows version 25.0 (IBM, Armonk, NY, USA). The normality of distribution of continuous variables was investigated using the Shapiro–Wilk test. Descriptive statistics were expressed as means and standard deviations for normally distributed variables. The Mann–Whitney U test was applied in the comparison of two dependent, non-normally distributed groups, and the paired sample t-test in the comparison of two independent, normally distributed groups. Statistical significance was set at a two-sided p value lower than 0.05.

## Results

The records of 229 patients who underwent coronary artery bypass surgery between January 2015 and March 2020 were reviewed. Five patients were found to have been admitted under emergency conditions, with three patients having a history of cerebrovascular disease. The records of 221 patients who met the study criteria were thus finally subjected to retrospective analysis. Demographic data and operational characteristics are shown in Table 1. The distribution consisted predominantly of males. In terms of comorbid diseases, the most common accompanying disease was hypertension.

**Table 1:** Demographic data and operation characteristics (number of patients/mean values ± SD)‬‬

Gender (M/F)	191/30
Age (years)	61.8 ± 11.1‬‬‬‬‬‬‬‬‬‬‬‬‬‬‬‬‬‬‬‬‬‬‬‬‬‬‬‬‬‬‬‬‬‬‬‬‬‬‬‬‬‬‬‬‬‬‬
BMI	28.9 ± 3.8
‬‬‬‬‬‬‬‬‬‬‬‬‬‬‬‬‬‬‬‬‬‬‬‬‬‬‬‬‬‬‬‬‬‬‬‬‬‬‬‬‬‬‬‬‬‬‬‬‬‬‬‬‬‬‬‬‬‬‬‬‬‬‬‬‬‬‬‬‬‬‬‬‬‬‬‬‬‬‬‬‬‬‬‬‬‬‬‬‬‬‬‬‬‬‬‬‬‬‬‬‬‬‬‬‬‬‬‬‬‬‬‬‬‬Smoking status ( ±) [%]	‬‬‬‬‬‬‬‬‬‬‬‬‬‬‬‬‬‬‬‬‬‬‬‬‬‬‬‬‬‬‬‬‬‬‬‬‬‬‬‬‬‬‬‬‬‬‬‬‬‬‬‬‬‬‬‬‬‬‬‬‬‬‬‬‬‬‬‬‬‬‬‬‬‬‬‬‬‬‬‬‬‬‬‬‬‬‬‬‬‬‬‬‬‬‬‬‬‬‬‬‬‬‬‬‬‬‬‬‬‬‬‬‬‬94/127 [42,5%]
Diabetes mellitus ( ±) [%]	129/125 [58,3%]
Hypertension ( ±) [%]	182/39 [82,3%]
COPD ( ±) [%]	54/167 [24,4%]
Preoperative LVEF (%, Mean)	52.1 ± 11

*LVEF left ventricle injection fraction, COPD chronic obstructive pulmonary disease*

The operational characteristics are listed in Table 2. The low use of red blood cells was particularly noteworthy. None of the patients received balloon pump support, but 20 were transferred to the CICU while intubated. The anesthesia methods applied in cases transferred to intubation intensive care were examined, revealing that regional anesthesia was used in two patients, but not in the other 18. Examination of the extubation times of the two patients who received regional anesthesia and were then transferred to intubation intensive care showed that the first was extubated at 20 min (the earliest time among the patients who were intubated), and the other after 45 min (when calculating the extubation time in the intensive care unit, the time at the end of the operation was taken as zero). When analgesia methods were compared among the patients transferred to the CICU, extubation time was significantly earlier in those receiving regional anesthesia (p < 0.05). Analysis of the analgesia employed among patients transferred to the CICU revealed that a significantly higher number of patients were not given regional anesthesia (p < 0.05).

**Table 2:** Operative characteristics

Operative time (min)	182.1 ± 41.2
Red blood cell suspension (units)	0.2 ± 0.5
Fresh frozen plasma (units)	2.1 ± 1.1
Intra-aortic balloon pump	0
Extubation in the operating room ( ±) [%]	201/20 [90,04%]
Extubation time in the CICU (min)	89.9 ± 56

*CICU cardiovascular intensive care unit*

Anesthesia was induced with propofol and fentanyl. In terms of fentanyl dosages, 157 patients received 50 µg, 21 received 75 µg, and 43 patients received 100 µg. There was no difference in terms of the regional anesthesia techniques. Inhalation anesthesia was employed in all cases, and remifentanil was used for intraoperative analgesia. No difference was observed in terms of the remifentanil doses.

**Fig. 1:**
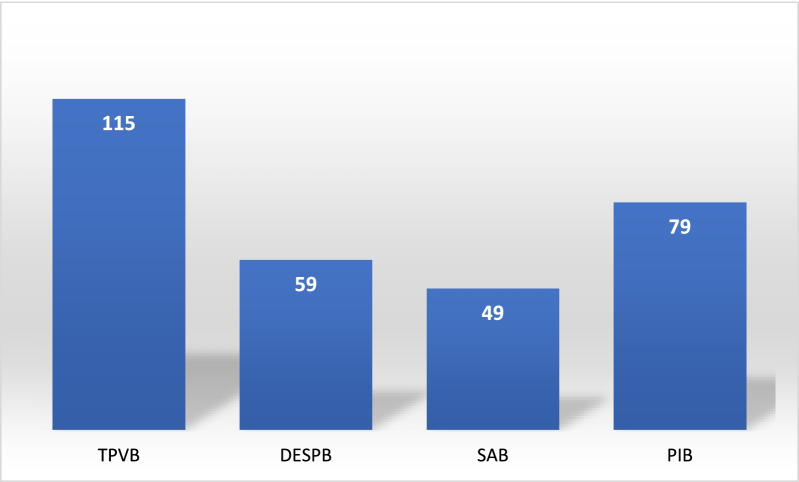
Regional analgesia techniques (numbers of patients). *TPVB: Thoracic paravertebral block, DIESPB: Dual-injection erector spinae plane block*. *SAB: Serratus anterior plane block, PIB: Parasternal intercostal block*

In terms of analgesia methods, regional analgesia was used in 179 cases. The distribution of the regional analgesia methods is shown in Fig. 1. The principal regional anesthesia techniques used were dual-injection erector spinae plane block (DIESPB) and thoracic paravertebral block (TPVB). In addition, serratus anterior plane block (SAB) and parasternal intercostal blocks were combined in only five cases.

Intraoperative opioid consumption is shown in Table 3. This was significantly low in cases in which regional anesthesia was used (p < 0.05).

**Table 3:** Opioid consumption during the intraoperative period (mean values ± SD)

Opioids	Regional anesthesia ( +) (n:179)	Regional anesthesia (−) (n:41)
Morphine (mg)	4.3 ± 1.8^*^	8.3 ± 1.8
Tramadol (mg)	33.2 ± 21.4^**+**^	115.3 ± 67.3
Pethidine (µg)	0	0

^*^p < 0.05 ^+^p < 0.05

**Fig. 2:**
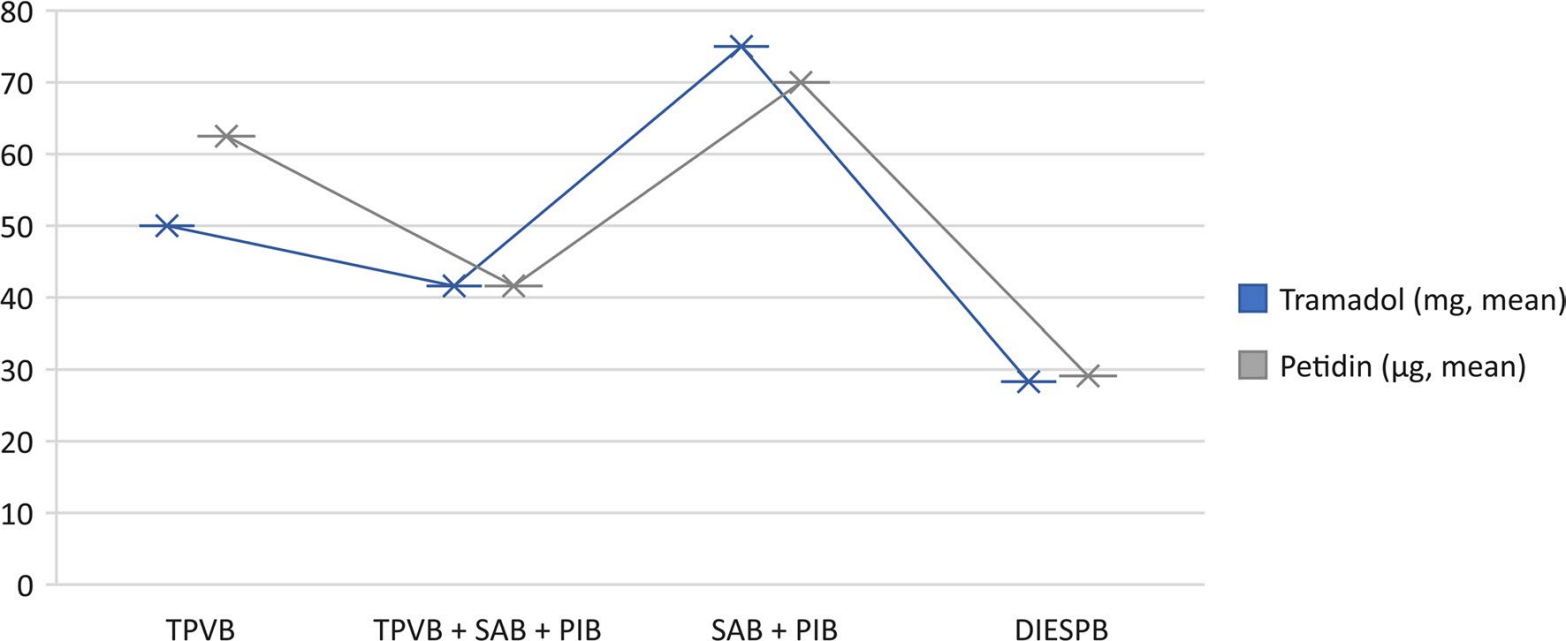
Opioid consumption according to fascial plane blocks. *TPVB: Thoracic paravertebral block, DIESPB: Dual-injection erector spinae plane block*. *SAB: Serratus anterior plane block, PIB: Parasternal intercostal block*

Opioid consumption after 24 h is shown in Table 4. Examination showed that based on the cardiovascular surgery and anesthesia clinics’ postoperative pain control protocol, in case of NRS scores above 4 during rest, the patient received intravenous tramadol 50 mg, while if the NRS remained above 4, intravenous 50 µg pethidine was also administered. After assessment based on this protocol, opioid consumption was significantly lower in patients undergoing regional analgesia. Analysis of opioid consumption in terms of regional analgesia techniques showed that patients who received DIESPB had lower opioid requirements (Table 5, Fig. 2).

**Table 4:** Opioid consumption in the first postoperative 24 h (mean values ± SD)

	Regional anesthesia ( +) (n:179)	Regional anesthesia (−) (n:41)
Tramadol (mg)	34.7 ± 31*	95.6 ± 44
Pethidine (µg)	20.6 ± 9.4^**+**^	45.6 ± 28.2

*Tramadol 50 mg was administered if the NRS score was* > *4. If the NRS score was above 4 despite the administration of tramadol, pethidine 50 µg was administered*

^*^p < *0.05* ^+^ p < 0.05

**Table 5:** Opioid consumption in fascial plane blocks

	Tramadol (mg, mean)	Pethidine (µg, mean)
TPVB	50 ± 28.8^#^	62.5 ± 29.7^**#**^
TPVB + SAB + PIB	41.6 ± 18.1^**+**^	41.6 ± 27.6^**+**^
SAB + PIB	75 ± 25	70 ± 24.4
DIESPB	28.3 ± 22.6^*^	29.1 ± 24.6^*^

*TPVB thoracic paravertebral block, DIESPB dual-injection erector spinae plane block, SAB serratus anterior plane block, PIB Parasternal intercostal block*

^*^p < 0.01 (DIESPB compared to other blocks) ^+^p < 0.05 (TPVB + SAB + PIB blocks compared to SAB + PIB blocs)

^#^p < 0.05 (TPVB compared to SAB + PIB blocks)

Analysis of the patients’ starting times for receipt of breathing exercises showed that all began at the postoperative 6th hour, according to the intensive care follow-up forms. No difference was observed in TriFlo deep breathing start times among the patients who underwent regional anesthesia. Length of stay in the ICU, time to first mobilization, and time to discharge were significantly lower among the patients receiving regional anesthesia (p < 0.05). No positive contribution of regional anesthesia, a recovery criterion, was observed at discharge. (Table 6).

**Table 6:** Fast track recovery criteria

	All patients (N:221)	RA ( +) (N:179)	RA (−) (N:41)
Length of stay in CICU (h) (m ± SD)	22.7 ± 6.9	21.8 ± 6.4^*^	26.5 ± 7.8
First mobilization time (h) (m ± SD)	20.3 ± 6.5	19.6 ± 6.2^**+**^	24.2 ± 7.6
Time to discharge (d) (m ± SD)	4.7 ± 1.9	4.7 ± 2	4.9 ± 1.5

*CICU cardiovascular surgery intensive care unit, RA regional anesthesia, N number*

*h hour, d day, m mean, SD standard deviation*

^*^p < *0.05 Length of stay in CICU is shorter in patients undergoing regional analgesia*

^+^*p* < *0.05 First mobilization time is shorter in patients undergoing regional analgesia*

Examination of postoperative respiratory complications revealed no cases associated with regional anesthesia. Only two patients experienced postoperative atelectasis, but none required postoperative non-invasive or invasive mechanical ventilation support.

## Discussion

Ultrasound-guided interfascial plane blocks are a recent development in modern regional anesthesia practice and constitute a new route of transmission for local anesthetics to various anatomical locations [7]. Fascial plane blocks are frequently used in surgery, with successful results and growing popularity being reported. However, only the passage of time and accumulating scientific evidence will show whether or not they represent an alternative or primary option.

Analgesia in cardiac surgery traditionally relied on large doses of intravenous (IV) opioids. However, this practice has changed due to “fast-tracking” or the expectation of tracheal extubation shortly after admission to the ICU. Regional anesthetic techniques may help reduce acute postoperative pain and the potential development of chronic pain by reducing sensitization from noxious surgical injury and opioid-induced hyperalgesia. The use of neuraxial techniques in cardiac surgery with full heparinization and hemodynamic instability remains controversial. Fascial plane chest wall blocks are growing in popularity as an alternative, owing to their simplicity and perceived low complication risks, when thoracotomy or sternotomy are required. The wider implementation of minimally invasive surgical approaches over the last two decades and novel techniques in ultrasound-guided regional anesthesia have meant that it is now not unusual to achieve intraoperative conditions permitting extubation in the operating room [8, 9]. Regional anesthesia has long been used in cardiac surgery in our clinic. The principal goals are to reduce opioid consumption, facilitate extubation in the operating room, and reduce opioid side-effects. We routinely employ fascial plane blocks and are satisfied with their results in cardiac surgery. Our pain management results are consistent with those reported in the available literature.

The thoracic intercostal nerves (T1–T11) are primarily responsible for sensory innervation of the chest wall. Each spinal nerve exits an intervertebral foramen and divides into a dorsal and ventral ramus, which communicates with the sympathetic trunk via the white and gray rami. The dorsal rami supply the muscles, bones, joints, and skin of the mid-back. The ventral rami run together with blood vessels, initially between the pleura and endothoracic fascia, and then between the internal and innermost intercostal muscles, innervating the lateral and anterior chest walls. The medial (C8–T1) and lateral (C5–C7) pectoral, long thoracic (C5–C7), and thoracodorsal (C6–C8) nerves originate from the brachial plexus for motor innervation to the muscles of the chest wall, but also carry sensory nerve fibers [10]. Innervation of the chest wall is essential for effective analgesia. Fascial plane blocks that provide maximal cover of the innervation play a key role in the achievement of effective outcomes.

The benefits of neuraxial blockade include lower opioid requirements, lower pain scores, fewer pulmonary complications, lower incidences of stroke and myocardial ischemia, less vestigial stress response, a lower incidence of kidney failure, lower infection rates, shorter ICU stays, lower anesthesia costs, and earlier discharge. Peripheral blocks contribute significantly to hemodynamic stability, high safety profiles, opioid sparing, and improved cough [6, 11, 12]. Patients receiving oral anticoagulants and antiplatelet drugs, two medications frequently employed in cardiac surgery, are at an increased risk of hematoma. Fascial plane blocks have becoming increasingly popular compared to central blocks in cardiac surgery in recent years.

Fascial plane block selection is important for analgesia in all surgeries. Correct fascial plane block, surgical selection, and multimodal analgesia management are the most important steps toward success. Various blocks are employed for postoperative pain reduction after cardiac surgery, including bilateral pectoralis I/II (PEC I/II), erector spinae (ESB), serratus anterior plane (SAPB), and paravertebral (PVB) blocks [9]. The cardiothoracic literature regarding the use of PECS block is currently limited to case reports. Kaushal et al. [13] described PECS block as more effective than intercostal block in postoperative analgesia studies in pediatric cardiac surgeries performed with thoracotomy.

The PECS II block is used when a local anesthetic is injected between the pectoralis minor and serratus anterior muscles, typically along the fourth rib. It blocks the anterior divisions of the thoracic intercostal nerves, long thoracic nerve, and thoracodorsal nerve [14]. In a 2018 randomized trial of 40 adult patients undergoing cardiac surgery. Kumar et al. [12] found that patients receiving bilateral PECS block reported lower pain scores at rest and with coughing, plus lower rescue pain medication requirements compared to controls without blocks. In that study, the authors added local anesthetic infiltration around the drain in addition to the PECS block. The results of the PECS block group may therefore not be solely attributable to the fascial plane block. Moreover, the fact that very few patients had VAS scores higher than 4 in the no-block group can be interpreted differently in terms of patient pain thresholds and the study results. Anterolateral analgesia of the thoracic wall is essential in order to achieve good results in cardiac surgery. However, it seems difficult to achieve this with the PECS block, although the technique may be considered for multimodal analgesia. Although it seems difficult to achieve this with PECS block, it can be considered for multimodal analgesia. It may also be effective in reducing opioid consumption even if it does not provide sufficient analgesia. The serratus anterior plane block (SAPB) is an ultrasound-guided technique for the axillary region, and is more lateral and posterior than PEC blocks. Local anesthetic is injected in the plane between the serratus anterior and latissimus dorsi muscles, where the intercostobrachial nerve, lateral cutaneous branches of the intercostal nerves (T3–T9), long thoracic nerve, and thoracodorsal nerve are located [6, 15]. It covers a larger posterolateral area than the PECS block. A limited number of studies have found that both PECS and SAP blocks are equally effective in providing predictable postoperative analgesia and can be used for postoperative analgesia in cardiac surgery [13, 15]. More effective results have been obtained in cardiac surgery with thoracotomy. This increases the effect of TPVB when added, and has been shown to reduce opioid consumption when combined with the parasternal intercostal block. However, the serratus plane and parasternal intercostal block were the least effective combinations compared with regional anesthesia techniques.

Parasternal nerve blocks target the anterior and posterior intercostal nerves located below each rib, lateral to the sternum. In order to perform a parasternal block, a local anesthetic is injected between the pectoralis major and external intercostal muscles. The anterior branches of the intercostal nerve penetrate these two muscles and innervate the internal mammary area. Infiltration of the local anesthetic in this area should therefore block the anterior branches of the intercostal nerves [6, 13, 16–18]. In cases in which an internal mammary artery graft is used, these muscles are dissected. We suspect that the spread of the local anesthetic in this block may be controversial. The parasternal intercostal block was not used by itself in this study, but has been shown to enhance the effect of TPVB when applied in addition to it. The technique produces ipsilateral, segmental, somatic, and sympathetic nerve blockade in contiguous thoracic dermatomes. This technique was first described in 1905 by Sellheim, and was reintroduced in clinical practice by Eason and Wyatt in 1978. The thoracic paravertebral space is filled with adipose tissue along with the intercostal nerve, artery, vein, and sympathetic trunk. The intercostal nerve enters the space medially and assumes a lateral position along the inferior edge of the rib [10, 19]. TPVBs are frequently used in cardiac surgery. El Shora et al. [20] concluded that TPVB is as safe and effective as thoracic epidural analgesia in relieving sternotomy pain after cardiac surgery, with fewer side-effects and a shorter ICU stay. Published studies have reported effective and hemodynamically stable results with TPVB. Although ultrasound guidance reduces the complication rates, it remains an invasive technique. TPVB should be performed for at least two levels, as well as bilaterally, for effective analgesia, since the technique yielded effective results in the present study. The results were better when additional blocks were added to the TPVB. A paravertebral block is a safer technique if performed with ultrasound, but is as reliable as fascial plane blocks. Avoiding central blocks with coagulation problems should be considered when using paravertebral blocks.

The ESPB is a fascial plane block performed by injecting local anesthetic deep into the fascial plane of the erector spinae muscle overlying the transverse processes of the thoracic vertebrae [21]. ESPBs have been found to be effective in cardiac surgery in numerous previous studies [3, 22, 23], although the mechanisms of action involved are controversial. Clinical and cadaveric studies provide information about the spread of local anesthetics. The most likely spread is among the fascial planes. Findings concerning the involvement of the ventral branches of the spinal nerves are inconsistent. Therefore, if we accept the involvement of the ventral rami, this block seems to represent an extension of the paravertebral area [27].

In addition to studies reporting effective results in cardiac surgery, others have suggested that ESPB is not effective for sternotomy pain [25].

ESPB does not relieve chest pain around the sternum during cardiac surgeries in our clinic. The first complaint among patients undergoing ESPB was severe pain around the sternum, and we did not therefore use classic ESPB on a frequent basis. We described different modifications of the ESPB for the purpose of increasing its transition to the paravertebral area. Using this technique, known as DIESBP, we observed a dramatic decrease in sternal pain during cardiac surgery [26, 27]. In the present study, DIESPB was more effective than the other regional anesthesia techniques.

In conclusion, fascial plane blocks reduce opioid consumption. Different fascial plane blocks are used in cardiac surgery, and published studies generally describe effective results. Analgesic methods that allow extubation in the operating room are considered effective in cardiac surgery. The most important issue is selecting the appropriate fascial plane block. The major concern with fascial plane blocks is that they have different outcomes. Different effects from the same block can be observed in one type of operation performed by the same practitioner. Fascial planes must be well understood. The deep fasciae of the chest wall (pectoral region) are usually formed by a single layer of undulated collagen and elastic fibers adhering to the underlying muscles. The presence of varying numbers sublayers in the thoracolumbar fascia has been previously described. The middle thoracolumbar fascia contains three sublayers. It is not usually possible to determine the number of fascial layers, and to clearly differentiate between them, using current ultrasound technology. It is also still unclear whether there is an optimal choice of layer for local anesthetic injection, or whether this will affect the spread of medication or clinical outcomes [7, 28]. This may alter the effectiveness of fascial plane blocks. Although local anesthetics sometimes spread well under ultrasound in fascial plane blocks, they may sometimes not exhibit the outcomes expected for clinical efficacy. This is still unclear because local anesthetics do not spread between the correct fascial planes, and because this cannot be fully determined under ultrasound. We suspect that the importance of fascial plane blocks will gradually improve rapid recovery and effective analgesia in cardiac surgery in the future.

## Conclusion

Regional anesthesia techniques significantly reduce opioid consumption during cardiac surgery. The importance of fascial plane blocks in terms of rapid healing criteria in cardiac surgery is growing rapidly. Fascial plane blocks occupy a particularly important place in terms of significantly reducing opioid consumption and also supporting extubation in the operating room. According to the study's findings, DIESBP was the fascial plane block providing the most effective analgesia in cardiac surgery We think more extensive, randomized, controlled studies are needed to determine the most effective techniques in terms of fascial plane blocks.

## Limitations

The principal limitation of this study lies in its retrospective nature. The patients’ pain scores were determined using a uniform scale. Pain follow-ups were carried out by intensive care and pain nurses. The commencement of the breathing exercises was based on the operability of the device, patient compliance, and nursing support. Mobilization and length of ICU stay are recovery criteria, although it is controversial whether or not they determine early recovery.

## Data Availability

The study data are recorded in the hospital information system. The data, from which all identifying information has been removed, are available from the corresponding author, Sami Kaan Coşarcan.

## Abbreviations

ICU: Intensive care unit
TEA: Thoracic epidural analgesia
CABG: Coronary artery bypass grafting
CICU: Cardiovascular intensive care unit
NRS: Numerical rating scale
DIESPB: Dual-injection erector spinae plane block
TPVB: Thoracic paravertebral block
SAB: Serratus anterior plane block
ESPB: Erector spinae plane block
PECS: Pectoralis block

## References

[1] Bigeleisen PE, Goehner N (2015). Novel approaches in pain management in cardiac surgery. Curr Opin Anaesthesiol.

[2] Doehring A, Oertel BG, Sittl R (2013). Chronic opioid use is associated with increased DNA methylation correlating with increased clinical pain. Pain.

[3] Nagaraja PS, Ragavendran S, Singh NG (2018). Comparison of continuous thoracic epidural analgesia with bilateral erector spinae plane block for perioperative pain management in cardiac surgery. Ann Cardiac Anaesthesia.

[4] Engelman DT, Ali WB, Williams JB (2019). Guidelines for perioperative care in cardiac surgery: enhanced recovery after surgery society recommendations. JAMA Surg.

[5] Bignami E, Landoni G, Biondi-Zoccai GG (2010). Epidural analgesia improves outcome in cardiac surgery: a meta-analysis of randomized controlled trials. J Cardiothorac Vasc Anesth.

[6] Liu H, Emelife PI, Prabhakar A (2019). Regional anesthesia considerations for cardiac surgery. Best Pract Res Clin Anaesthesiol.

[7] Elsharkawy H, Pawa A, Mariano ER (2018). Interfascial plane blocks: back to basics. Reg Anesth Pain Med.

[8] Rivat C, Bollag L, Richebé P (2013). Mechanisms of regional anaesthesia protection against hyperalgesia and pain chronicization. Curr Opin Anaesthesiol.

[9] Noss C, Prusinkiewicz C, Nelson G (2018). Enhanced recovery for cardiac surgery. J Cardiothorac Vasc Anesth.

[10] Kelava M, Alfirevic A, Bustamante S (2020). Regional anesthesia in cardiac surgery: an overview of fascial plane chest wall blocks. Anesth Analg.

[11] Chaney MA (1997). Benefits of neuraxial anesthesia in patients undergoing cardiac surgery. J Cardiothorac Vasc Anesth.

[12] Kumar KN, Kalyane RN, Singh NG (2018). Efficacy of bilateral pectoralis nerve block for ultrafast tracking and postoperative pain management in cardiac surgery. Ann Card Anaesth.

[13] Kaushal B, Chauhan S, Saini K (2019). Comparison of the efficacy of ultrasound-guided serratus anterior plane block, pectoral nerves ii block, and intercostal nerve block for the management of postoperative thoracotomy pain after pediatric cardiac surgery. J Cardiothorac Vasc Anesth.

[14] Yu S, Valencia MB, Roques V (2019). Regional analgesia for minimally invasive cardiac surgery. J Card Surg.

[15] Moll V, Maffeo C, Mitchell M (2018). Association of serratus anterior plane block for minimally invasive direct coronary artery bypass surgery with higher opioid consumption: a retrospective observational study. J Cardiothorac Vasc Anesth.

[16] Chaudhary V, Chauhan S, Choudhury M (2012). Parasternal intercostal block with ropivacaine for postoperative analgesia in pediatric patients undergoing cardiac surgery: a double-blind, randomized, controlled study. J Cardiothorac Vasc Anesth.

[17] Lee CY, Robinson DA, Johnson CA (2019). A randomized controlled trial of liposomal bupivacaine parasternal intercostal block for sternotomy. Ann Thorac Surg.

[18] Ohgoshi Y, Ino K, Matsukawa M (2016). Ultrasound-guided parasternal intercostal nerve block. J Anesth.

[19] Krediet AC, Moayeri N, van Geffen GJ (2015). Different approaches to ultrasound-guided thoracic paravertebral block: an illustrated review. Anesthesiology.

[20] El Shora HA, El Beleehy AA, Abdelwahab AA (2020). Bilateral paravertebral block versus thoracic epidural analgesia for pain control post-cardiac surgery: a randomized controlled trial. Thorac Cardiovasc Surg.

[21] Forero M, Adhikary SD, Lopez H (2016). The erector spinae plane block: a novel analgesic technique in thoracic neuropathic pain. Reg Anesth Pain Med.

[22] Macaire P, Ho N, Nguyen T (2019). Ultrasound-guided continuous thoracic erector spinae plane block within an enhanced recovery program is associated with decreased opioid consumption and improved patient postoperative rehabilitation after open cardiac surgery-a patient-matched, controlled before-and-after study. J Cardiothorac Vasc Anesth.

[23] Krishna SN, Chauhan S, Bhoi D (2019). Bilateral erector spinae plane block for acute post-surgical pain in adult cardiac surgical patients: a randomized controlled trial. J Cardiothorac Vasc Anesth.

[24] Chin KJ, El-Boghdadly K (2021). Mechanisms of action of the erector spinae plane (ESP) block: a narrative review. Can J Anaesth.

[25] Taketa Y, Irisawa Y, Fujitani T (2018). Ultrasound-guided erector spinae plane block elicits sensory loss around the lateral, but not the parasternal, portion of the thorax. J Clin Anesth.

[26] Coşarcan SK, Gürkan Y, Doğan AT (2020). Targeted modification of erector spinae plane block. Acta Anaesthesiol Scand.

[27] Coşarcan SK, Doğan AT, Gurkan Y (2021). Analgesic effect of dual injection technique for the erector spinae plane block in beating heart coronary by-pass surgeries. Cureus.

[28] Stecco C, Macchi V, Porzionato A (2011). The fascia: the forgotten structure. Ital J Anat Embryol.

